# Micropropagation of Bioencapsulation and Ultrastructural Features of Sainfoin (*Onobrychis viciifolia*) Grown *In Vivo* and *In Vitro*

**DOI:** 10.1155/2014/680356

**Published:** 2014-06-19

**Authors:** Sadegh Mohajer, Rosna Mat Taha, Minoo Mohajer, Arash Khorasani Esmaeili

**Affiliations:** ^1^Institute of Biological Sciences, Faculty of Science, University of Malaya, 50603 Kuala Lumpur, Malaysia; ^2^Department of Plant Biology, Faculty of Biological Sciences, Kharazmi University, Tehran 15719-14911, Iran

## Abstract

To explore the potential of *in vitro* rapid regeneration, three varieties (Golpaygan-181, Orumieh-1763, and Gorgan-1601) of sainfoin (*Onobrychis viciifolia* Scop. syn. *Onobrychis sativa* L.) were evaluated. For the first time, an encapsulation protocol was established from somatic embryogenic callus in torpedo and cotyledonary stages to create artificial seeds. Callus derived from different concentrations of Kinetin (0–2.0 mg L^−1^) and Indole-3-acetic acid (0–2.0 mg L^−1^) was coated with sodium alginate and subsequently cultured either in Murashige and Skoog (MS) medium or in soil substrate. Adventitious shoots from synthetic beads developed into rooting in full and half strength MS medium supplemented with various concentrations of auxin and cytokinin. Prolonged water conservation of black and red soils (1 : 1) had the highest rate of survival plantlets in the acclimatization process. Diverse resistance techniques in *Onobrychis viciifolia* were evaluated when the plants were subjected to water deficiency. Higher frequency of epicuticular waxes was observed in *in vivo* leaves compared to *in vitro* leaves. Jagged trichomes nonsecreting glands covered by spines were only observed in the lower leaf side. Ultimately, stomata indices were 0.127 (abaxial), 0.188 (adaxial) in *in vivo* and 0.121 (abaxial), 0.201 (adaxial) in *in vitro* leaves.

## 1. Introduction

Despite the fact that sainfoin (*Onobrychis viciifolia*) is an important forage species, it has received little attention and assessment for* in vitro* studies. Among the attributes of sainfoin, it improves soil fertility, where the environmental conditions limit the cultivation of alfalfa, and produces safe bloat forage. Therefore, the progress of this species by genetic engineering techniques will contribute significant advantages for plant breeding objectives.

A basic prerequisite of genetic engineering is advance of an efficient adventitious shoot regeneration system for the desired species. Rapid multiplication of shoot tips is notable to reduce the cost and genetic purity of micropropagated plants. Indeed, different auxins and cytokinins concentrations in MS medium play an important role in achieving a desired rate of multiple shoot formation. Ratio of regeneration depends on culture type, composition of the medium, and the variety used [1].

Plant regeneration via somatic embryogenesis is usually investigated for important objectives of somaclonal propagation and multiplication in particular genetic transformation. In reality, the critical conversion of somatic embryos into plants is attained through maturation and germination stages [2, 3]. Nowadays, production of artificial seeds or synthetic seeds, consisting of enclosed somatic embryos or shoot buds, is a highly common propagation technique. This system is an outstanding proficiency used to propagate and preserve plants and assess many species for microshoots production from somatic embryogenesis [4]. This facile and unique propagation system deliberated by Bapat et al. [5] can be utilized on both difficult to root species and worthwhile varieties. In order to promote root induction of sainfoin and overcoming the effect of cytokinin hormones during rooting, two auxin solutions, 1-naphthaleneacetic acid (NAA) and indole-3-butyric acid (IBA), with different concentrations have been suggested [6, 7].

*In vivo* cultivation is totally different compared to* in vitro* growth culture during the acclimatization process. Relative humidity (RH) is an important criterion that promotes the morphological, physiological, and biochemical features of plantlet when plantlets are transferred to* in vivo* condition for acclimatization [8, 9]. Moreover, nutrient retained in* in vitro* leaves is another important factor in the process of acclimatization [10]. The exclusive novelty of the current study is successful* in vitro* regeneration from synthetic seeds coated consisting of embryogenic callus, while previous studies evaluated the adventitious shoot regeneration from a range of explants, including mature [11] and immature embryos, root, leaf, and stems [12].

Wide varieties of plant microstructures, including light reflection and water absorption structures, have been already defined by the scanning electron microscope (SEM). The most significant threat in plant life can be referred to high temperature, because of intense radiation and decrement of water loss as a growth limiting factor. Physiological activity of land plants mainly depends on conservation of water which is carried out via plant roots. In order to hold the water and avoid the filtration of ions from interior structure in plants, a protective waxy layer called cuticle is developed that covers the epidermis cells from inside the plant [13, 14].

Whilst the intracuticular waxes are the main transport barrier to prevent the water loss and leach the molecules from inside of the living cells [15, 16], the epicuticular waxes have also an outstanding role in different plants as an interface layer. Epicuticular waxes are the cause of irritability control, self-cleaning [17], sliding of insects [18], reflection of visible light, absorption of UV radiation [19, 20], and adhesion reduction of particles [21]. Water loss might be influenced by trichomes function and affected on surface wettability [22].

In the present study, short-term stability and regeneration capacity of synthetic seeds containing embryogenic callus of* Onobrychis viciifolia* were investigated. Comparison of intact (*in vivo*) and* in vitro* leaf morphological structures based on epicuticular waxes, convex cells, and trichomes was also carried out using scanning electron microscope (SEM). Ultimately, this study suggests that evaluation of different resistance strategies of intact plant can be analyzed against water loss.

## 2. Materials and Methods

### 2.1. Explant Source

Seeds of three superior varieties (Golpaygan-181, Orumieh-1763, Gorgan-1601) of* Onobrychis viciifolia* were selected from the natural resources existing of gene bank in Iran. The best sterilization procedure of sainfoin's seeds was achieved when 50% Chlorox (outside the laminar chamber-1 min) and 70% of alcohol (inside the laminar chamber-1 min) were treated, respectively. After a couple of weeks, all seeds were almost germinated in the Murashige and Skoog medium (MS) supplemented with 3% (w/v) sucrose and 0.75% (w/v) agar. Explant sources were derived from aseptic seedlings to leaf, stem, and root segments.

### 2.2. Embryogenic Callus Induction

After inoculation of seeds in MS medium, stem and leaf explants of sainfoin were cut into small pieces (2-3 mm) from aseptic seedlings using a sharp sterile blade. To induce the callus, prepared explants were inoculated in MS medium fortified with different concentrations of Kinetin (0–2.0 mg L^−1^) and Indole-3-acetic acid (0–2.0 mg L^−1^). All explants were placed in culture room at 25 ± 1°C, 70% humidity and 16 h light photoperiod provided by cold fluorescent lamps for 3 weeks. Double staining method was used to ensure that the callus has truly regeneration capacity and contains the embryonic cells [23]. Fresh weight and percentage of produced callus from leaf and stem explants were measured. Different callus textures (compact and friable) were also evaluated after 3 weeks. Five various stages of somatic embryogenesis were observed using Dinocapture camera. In addition, stem and leaf explants produced adventitious shoots directly in some hormone concentrations which were calculated from 30 explants.

### 2.3. Encapsulation of Embryogenic Callus

Fresh calluses were collected in torpedo and cotyledonary stages of somatic embryogenesis from leaf and stem explants of the three sainfoin varieties (Gorgan-181, Orumieh-1763, and Gorgan-1601). Embryogenic calluses were isolated and mixed with/without 1 mg L^−1^ 6-benzylaminopurine (BAP) of autoclaved sodium alginate (5%) prepared from MS solution after adjusting the pH to 5.8. Then, the samples (3–5 mm) were dropped into solution of CaCl_2_·2H_2_O (1% w/v). Subsequently, the beads were retained in CaCl_2_·2H_2_O solution for 30 min and transferred to distilled water after the incubation period.

### 2.4. Germination Medium/Substrate

The beads (without BAP) of three varieties were germinated on various media and substrates: (1) MS basal medium + 3% sucrose + 0.8% agar (MSO) as control, (2) MS + 3% sucrose + 0.8% agar + 1 mg L^−1^ IBA, (3) MS + 3% sucrose + 0.8% agar + 1 mg L^−1^ NAA, (4) black sterilized soil (50% white peat + 50% black peat + 1.0 kg NPK fertilizer) + distilled water, (5) black nonsterilized soil + tap water. The beads with 1 mg L^−1^ BAP were cultured on MSO as well. All synthetic seeds were maintained in the culture room at 25 ± 1°C, 16 hours of light, and 8 hours of dark. The germination rate of artificial seeds was recorded after a couple of weeks. The beads were also cold-stored in the fridge at 4°C. Then, the beads were sown in MS basal medium for every 15-day interval to compare the viability of synthetic seeds.

### 2.5. Root Production and Acclimatization

Different auxins (NAA, IBA, and IAA) and cytokinin (BAP) concentrations were used to produce roots in regeneration process. Adventitious shoots (4-5 cm) obtained from synthetic seeds were transferred to full and half strength MS medium containing 3% sucrose and 0.75% agar. Cultures were preserved at 25 ± 1°C, 16 hours of light, and 8 hours of dark for one month. Then, the number of roots per shoot, callus percentage, microshoots, and dry weight of plantlets were recorded. The 4-week-old plantlets with well-developed roots were transferred to plastic pots containing different black and red (clay) soil combinations. Plantlets were maintained inside a growth chamber at 25 ± 1°C, 16-hour light for 2 weeks before being transferred to the greenhouse.

### 2.6. Scanning Electron Microscopy (SEM)

Leaf specimens of both* in vitro* and* in vivo* grown cultures were treated with the following solutions: 1 : 1 (v/v) glutaraldehyde (4%) and phosphate buffer solution at room temperature for 1 h, phosphate buffer solution and distilled water in 1 : 1 mixture for 30 min, and osmium tetroxide (4%) at 48°C for 14 h. After rinsing the samples with distilled water, the tissues were immersed in an ethyl alcohol series (10–100%) at 15 min intervals, followed by (1) 3 : 1 ethyl alcohol and acetone for 20 min, (2) 1 : 1 ethyl alcohol and acetone for 20 min, (3) 1 : 3 ethyl alcohol and acetone for 20 min, and (4) 100% acetone for 20 min. The final step was repeated four times. The replacement of acetone with carbon dioxide was carried out several times using a critical point dryer. Eventually, the samples were coated with gold for 1 min, before the observation by SEM (JEOL 6400).

Epidermal peel was evaluated to assess trichomes on the anticlinal walls, types of stomata, epicuticular waxes, convex cells, trichomes, and stomata index of the both abaxial and adaxial leaf surfaces.

Stomata index: (total numbers of stomata/(total numbers of epidermal cells + number of stomata)).

## 3. Results 

**Figure 1:**
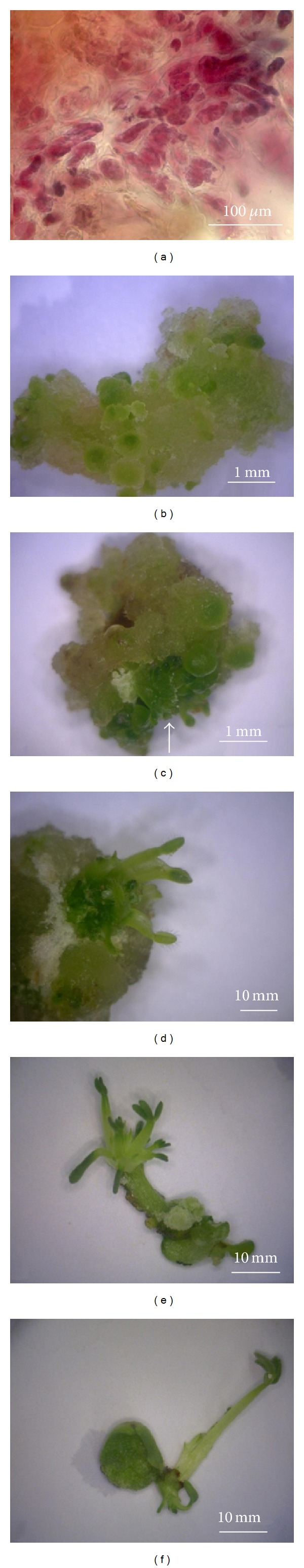
Stages of somatic embryogenesis in* Onobrychis viciifolia* (a–f). (a) Embryogenic callus in double staining method with camera lens, magnification of 40x; (b) globular stage and friable callus in root explants; (c) heart-shaped stage; (d) torpedo stage; (e) cotyledonary embryo stage; (f) shoot formation.

Leaf and stem explants of* O. viciifolia* were cultured in MS media supplemented with different concentrations of Kinetin and IAA. Double staining method was used to detect and differentiate the embryogenic from nonembryogenic callus. Embryonic cells had large nuclei with dense cytoplasms which were stained bright red with acetocarmine (Figure 1(a)). Generally, callus was formed in stem and leaf explants after 2-3 weeks, respectively. Two types of compact and friable callus were observed with cream, green, and light green colors. Best Kinetin and IAA concentrations were chosen based on the highest percentage and fresh weight of callus. Callus induction of three sainfoin varieties from stem and leaf explants is shown in Tables 1 and 2, respectively. Somatic embryos were enlarged into distinct bipolar structures and passed through typical developmental stages, including globular, heart, torpedo, and cotyledonary stages (Figures 1(b), 1(c), 1(d), and 1(e)). Although callus percentage was low in the control culture, MS medium supplemented with 1.5 mg L^−1^ Kinetin and 2 mg L^−1^ IAA had the highest percentage in both stem and leaf explants.

**Figure 2:**
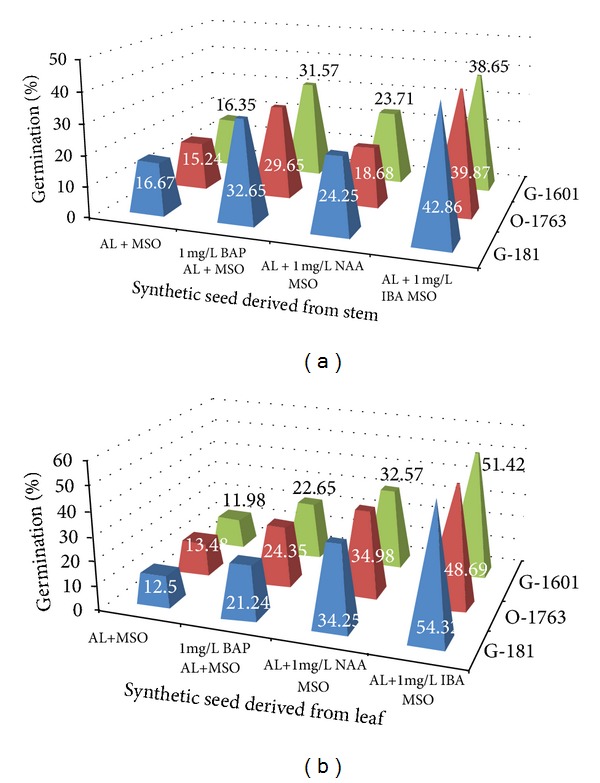
Percentage of germination from encapsulated somatic embryos of* Onobrychis viciifolia* on matrice of medium and variety: (a) stem and (b) leaf.

Pregerminated torpedo and cotyledonary shaped somatic embryos were used for encapsulation (Figure 1(d)). Encapsulated somatic embryos derived from stem explants induced the highest percentage of microshoots from the Golpaygan-181 variety. Conversion into adventitious shoots increased from the beads cultured in MSO (control culture) to MS medium supplemented with 1 mg L^−1^ NAA. However, MS medium supplemented with 1 mg L^−1^ IBA had an optimum effect on germination rate of synthetic seeds (Figure 2(a)). The survival rate of plantlets increased significantly when the beads derived from leaf explants were cultured in MS medium supplemented with 1 mg L^−1^ IBA. In line with this, 1 mg L^−1^ BAP and 1 mg L^−1^ NAA had also the positive influence in germination rates of the synthetic seeds, respectively. The survival rates of plantlets varied from 11.98% to 54.32% in the three sainfoin varieties, with maximum survival rate of beads obtained from leaf explants, which was observed in MS medium supplemented with 1 mg L^−1^ IBA in Golpaygan-181 variety (Figure 2(b)).

Sterilized soil showed the least preferred germination substrate in both stem and leaf synthetic seeds. In this regard, Golpaygan-181 had the maximum germination with 8.97% (Figures 3(b) and 3(d)). Although the survival rate was increased from control MS medium to nonsterilized soil in stem beads, soil substrates had a lower survival percentage in the leaf synthetic seeds (Table 3). Temperature and storage period are important factors to determine the regeneration frequency of the encapsulated somatic embryos. Approximately, 50–60% viability of stem and leaf synthetic seeds fell after 15-day storage at 4°C (Table 3).

Root production was a difficult stage after adventitious shoot induction in sainfoin synthetic seeds. MS medium supplemented with IBA showed no significant root production in this recalcitrant species. Concentrations of IBA and NAA with BAP did not induce high adventitious roots as well. Micropropagated shoots induced 52.62% root formation in half strength MS medium supplemented with 1 mg L^−1^ NAA. Shoot cultured on full MS medium supplemented with 1 mg L^−1^ NAA and 0.5 mg L^−1^ BAP had the highest percentage of rooting with 82.35% (Figure 3(e)). Among the three types of auxin, NAA was superior in comparison with IBA and IAA in terms of root number induction per shoot (Table 4).

The survival percentage of plantlets was affected by different soil substrates. It was revealed that black soil had lower efficiency as compared with red soil in acclimatization stage (Figure 3(f)). Combined substrates of red and black soils (ratio 1 : 1) had the highest survival percentage (98%) of plantlets (Table 5). Subsequently, plantlets were transplanted to the greenhouse with 100% survival rate and grown to 30 cm after 2 months.

**Figure 5:**
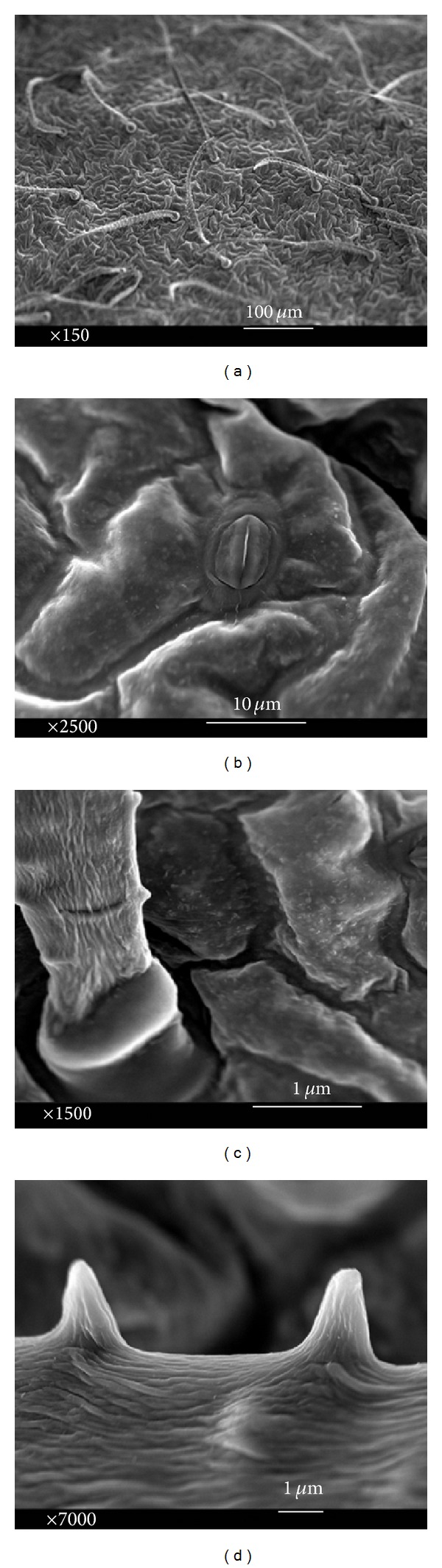
Abaxial side of* in vivo* leaf: (a) basic outlines of epidermal cells, (b) micropattern of small enhanced spots, formed by calcium oxide, (c) basal cell and stalk cell, and (d) folded jagged trichomes.

**Figure 6:**
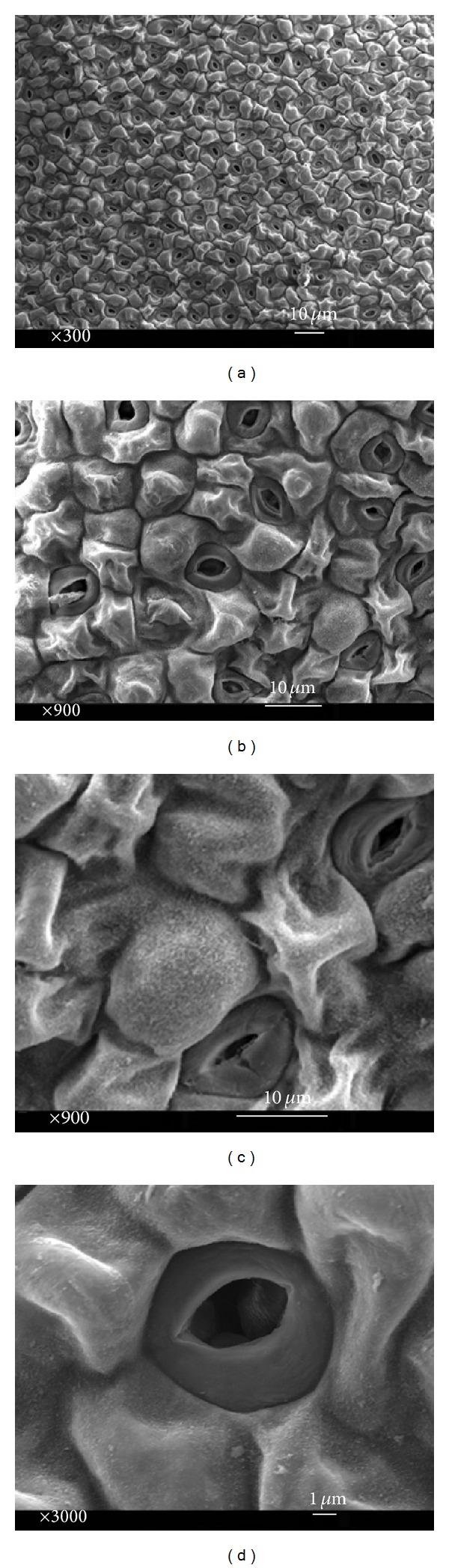
Adaxial side of* in vitro* leaf: (a) basic outlines of epidermal polygonal cells, (b) convex cell form with irregular cuticular shrinking, (c) guard cells and inner wall, and (d) open anomocytic stomata.

**Figure 7:**
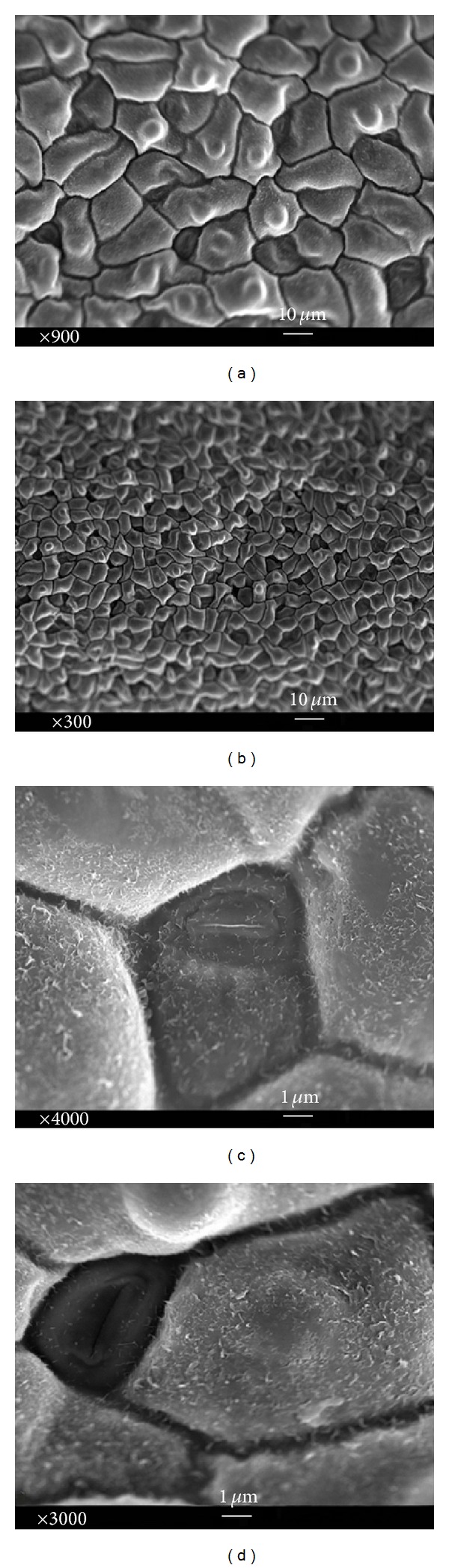
Adaxial side of* in vivo* leaf: (a) outlines of U-undulated and convex cell, (b) basic outlines of epidermal polygonal cells, (c) close anomocytic stomata, and (d) three-dimensional structures waxes.

Epidermal peels of* in vitro* and* in vivo* (intact plant) leaves were studied thoroughly. In order to achieve this, properties of both lower (abaxial) and upper leaf sides (adaxial) were assessed. Anticlinal walls and polygonal epidermal cells were exposed in the abaxial leaf sides of* in vivo* and* in vitro* (Figures 4(a) and 5(a)). Additionally, basic outline of the epidermal cells was elongated to polygonal cells with more than four edges, whereas cell boundaries were U-undulated in both* in vivo* and* in vitro* adaxial leaves (Figures 6(a) and 7(a)). Jagged trichomes nonsecreting glands covered by spines (botanically thorns) were only observed in the lower leaf side. Mean length of trichome in* in vitro* leaves was more than that in* in vivo* leaves (Figures 4(d) and 5(d)). Cuticle folding was induced by an undulated morphology of the underlying cellulose cell wall. Unlike the epidermal cells, folding or tubercular (verrucate) patterns were recognized in trichomes (Figures 4(d) and 5(d)). The cell sculptures or curvature of the outer epidermis wall (periclinal wall) has a great influence on the surface roughness in the micrometer scale.

Among the three basic forms of cell curvatures (tabular, convex, and concave), convex cells shaped cupolas were observed on the adaxial epidermal surface of both* in vivo* and* in vitro* leaves (Figures 6(b) and 7(b)). Some research has demonstrated that the impact of water loss leads to collapse cells. Along this line, sufficient humidity and water were the cause of convex cells shrinking in* in vitro* leaves (Figure 6(c)). Moreover, hierarchical surface structures including cuticle folding were not observed in the convex cells.

In the classification of wax morphologies, several three-dimensional structures such as crusts, threads, plates, platelets, filaments, rods, and tubules have been distinguished. Both* in vivo* and* in vitro* leaves had 3D and platelet waxes on their epidermal cells (Figure 7(c)). Platelet waxes on adaxial side were more than the underside of leaf in both growth cultures. In reality, the epidermal surface of* in vivo* leaves was exposed to a higher amount of platelet waxes in comparison with* in vitro* leaves in a specific pattern around stomata and subsidiary cells (Figures 5(c) and 7(c)).

Some microstructures of epidermal cells arising from subcuticular inserts of mineral crystals were identified in upper leaf side which were clear in intact specimens. In this manner, stomata and their surrounding cells had a micropattern of small enhanced spots, formed by subcuticular inserts of calcium oxalate (Figure 5(b)). To regulate both the water evaporation and gas exchanges, leaves developed specialized breathing pores called stomata which were anomocytic in this research (Figure 6(d)). Stomata indices were 0.127 (abaxial), 0.188 (adaxial) in* in vivo* and 0.121 (abaxial), 0.201 (adaxial) in* in vitro* leaves, respectively. Stomata interrupted the cuticular layer but could be closed (intact plants) when the humidity and water reduced in high temperature days of* in vivo* growth culture (Figures 5(b) and 7(d)). However, this barrier limits the uptake of carbon dioxide for photosynthesis from the atmosphere.

## 4. Discussion

Sainfoin seed production is not economical for farmers, since it should be harvested at flowering stage when the crop has the highest yield and best fodder quality. In order to overcome this situation, synthetic seeds technology might be the solution, due to the fact that the cost of seed production can be lowered through synthetic seed method compared to graining. Artificial seed induced through tissue culture is free from pathogens. Therefore, avoiding the bulk transportation of plants, quarantine, and spread of diseases are significant advantages of encapsulated propagules. In this study, either propagation of* Onobrychis viciifolia* was obtained in the large number or genetic uniformity of plants was preserved. Vegetative propagation method is recommended highly for preservation of uniformity and unique characteristics of sainfoin, while sexual propagation methods make heterogeneity varieties due to the outbreeding nature of this species [24].

Callus induction was achieved on MS medium supplemented with different concentrations of Kn and IAA. In this regard, the obtained embryogenic callus can be considered as the source of explants for further experiments. Encapsulation technique has sufficient potential for adventitious shoot production with high germination rate, which has been successfully applied in some species, like sandalwood,* Valeriana wallichii*,* Guazuma crinita*, and* Paulownia elongate* [25, 26].

Although a number of plants produced adventitious roots spontaneously in tissue culture, sainfoin lacks efficient root systems in* in vitro* culture. Therefore, rooting procedure from the shoot was carried out in a separate step by subculturing in full and half strength MS medium containing different concentrations of auxin and cytokinin. Root initiation process is critical mainly to provide sufficient stimulus by the concentration of required hormones. High doses of cytokinin used in the current study prevented the adventitious shoots for the normal rooting proliferation. The main restrictive parameters in vegetative propagation of sainfoin have been reported with very low frequency to be the establishment of rooted plantlets [6, 7]. Most of the previous researches used full strength MS medium supplemented with NAA to induce root [27, 28]. Besides, excised rooting from adventitious shoots was studied in both half and full strength MS medium in the present research. Subsequently, the highest rooting percentage was identified in full strength MS medium supplemented with 1 mg L^−1^ NAA and 0.5 mg L^−1^ BAP. Prolonged water conservation of red soil was significantly higher than black soil to increase the survival rates of plantlets in acclimatization. Deficit of water storage in black soil, which is required for further photosynthesis process, was inevitably compensated via the function of* in vivo* leaves. Since the black soil was lack of appropriate texture to maintain water, plantlet leaves were severely affected to wilting and necrosis of leaf blades. Despite the fact that plantlets indicated the notable survival rate in the red soil, equal mix ratio of black and red soil was recommended for acclimatization with 98% success rate.

It was observed that sainfoin beads were not cold resistant due to the low percentage of germination after storage at 4°C. In reality, other techniques should be contemplated to preserve the sainfoin artificial seeds from cold tension. Elvax 4260 (ethylene vinyl acetate acrylic acid terpolymer, Du Pont, USA) inoculation can be suggested to prevent the rapid water loss when the artificial beads are exposed to the surrounding environment [29].

Water transpiration is a natural and self-cooling mechanism for plants [30]. Subsequently, to minimize the water loss during drought season, leaves of nonsucculent plants use various mechanisms. In this respect, carbon dioxide absorption is raised and consequently photosynthesis process is enhanced due to the existence of stomata in amphistomatous (stomata on both sides) leaves [31]. To explore more clarifications, diverse resistance techniques used by* Onobrychis viciifolia* were evaluated when the plants were subjected to water deficiency. Transpiration barrier feature of leaves originates from hydrophobic material made up by a polymer called “cutin” and integrated and superimposed lipids called “waxes” [32, 33]. Additionally, to lessen the water deprivation, the cuticle prevents leaching of ions from inside the cells to outside. In fact, the cuticle and waxes are the main mentioned factors in transpiration barrier. Gibson [34] stated that thicknesses of both cuticle and waxes were increased in order to reduce the water loss in the dry regions. Similar observations in our research indicated that epicuticular waxes had higher frequency in* in vivo* leaves compared to* in vitro* leaves.

Convex cells morphology of microstructured surface which is found on the leaves and stems of flowering plants is originated by expansion of the outer side (periclinal wall) of the epidermis cells [35, 36]. Based on the comparison of* in vivo* and* in vitro* leaves, cells shrinking were induced by water loss due to convex cell morphology and sufficient water of MS media in* in vitro* growth culture (Figure 6(b)). Convex cells that contained water were observed in the epidermal layer of* in vivo* leaves when the water was scarce in soil (Figure 7(a)). Water evaporation rate was controlled by opening (Figure 6(b)) and closing (Figure 7(c)) functions of stomata. Closing of stomata took place in order to reduce the water evaporation when plants could receive insufficient amount of water through the roots (Figure 7(c)). Adversely, stomata were opened when the gas exchange process was the main objective (Figure 6(d)).

The functions of trichomes are to protect the plants from herbivores, heat, and sunlight. They also control leaf temperature as well as water loss through glandular trichomes. They produce various substances, which are stored at the plant surface. Moreover, leaf trichomes can protect plants against drought by reducing absorption of solar radiation, which in turn reduces the heat load and minimizes the need for transpirational cooling. Tolerance to drought can be also related to plant traits such as shoot and root morphology, root/shoot ratio, leaf wax production, the leaf area to volume ratio, and leaf area per stem [36, 37]. The cuticle folding of tissue was observed in the trichome (Figure 5(d)). Plant cuticle folds are used to (1) stabilize thin cell walls, (2) decrease wettability with water and contamination by both cuticle hydrophobicity and its microscopic sculpture (contact area reduction), and (3) set up surface reflection properties [38, 39]. Physiological and mechanical characteristics of species are influenced by bioactive elements such calcium [36]. Calcium oxalates are widespread in plants, including both dicotyledons and monocotyledons. They may represent storage forms of calcium and oxalic acid, and there has been some evidence of calcium oxalate resorption at times of calcium depletion. Fauteux et al. [40] reported the resistance increment of plants against pathogenic fungi by silica function. In the current study, calcium oxalates were clearly observed in intact leaves, but not in leaves of* in vitro* grown plants (Figure 5(b)).

## Figures and Tables

**Figure 3:**
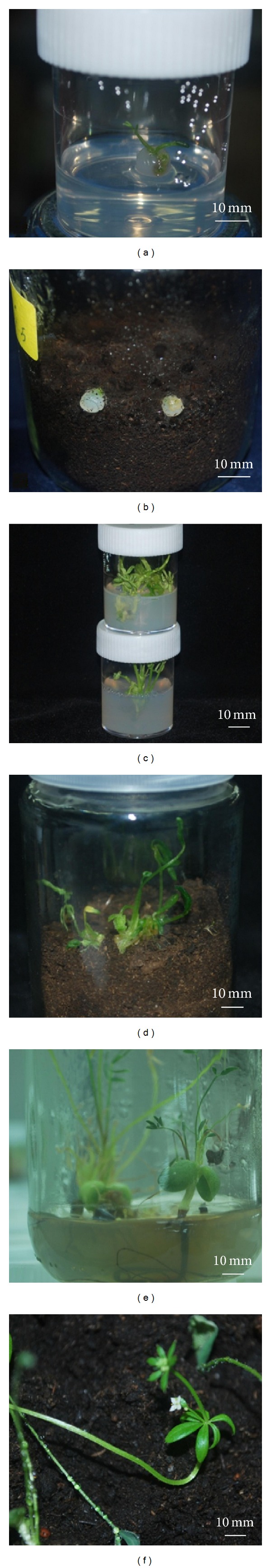
Micropropagation of* O. viciifolia*: (a) synthetic seeds in MS medium, (b) artificial seeds in black soil, (c) microshoots induction from synthetic seed, (d) seed germination in black soil, (e) adventitious roots induction, and (f) acclimatization and complete plantlets of* O. viciifolia*.

**Figure 3:**
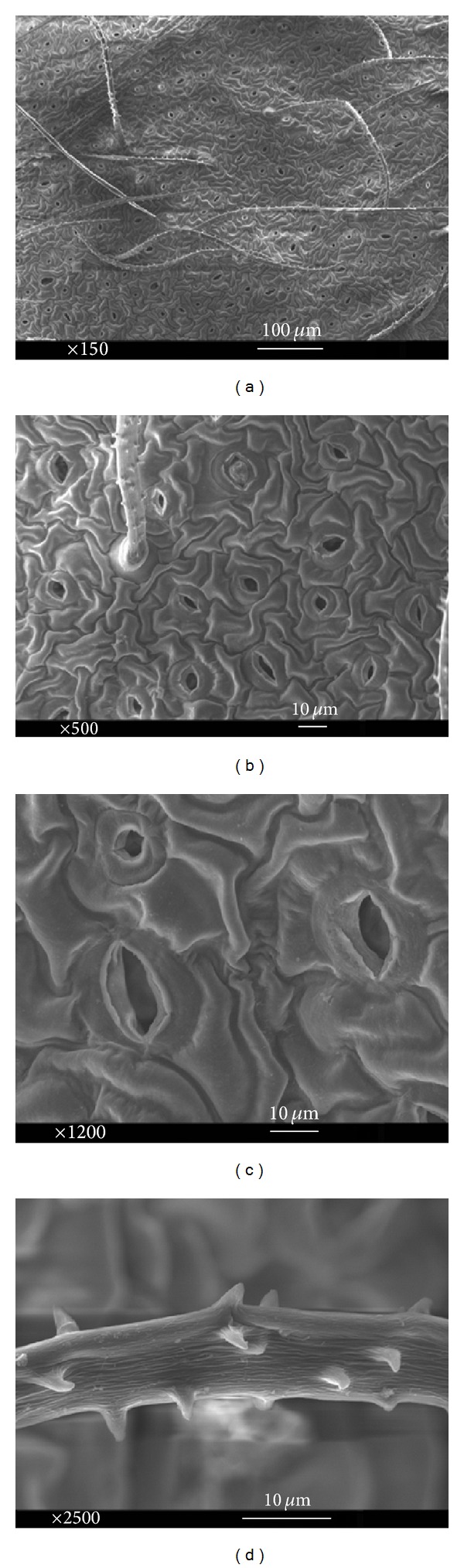
Abaxial side of* in vitro* leaf: (a) basic outlines of epidermal cells, (b) elongated polygonal cells with more than four edges, (c) open anomocytic stomata, and (d) folded jagged trichomes.

**Table 1:** Effect of Kinetin and IAA on mean weight and callus percentage of three *O. viciifolia* varieties: Golpaygan-181, Orumieh-1763, and Gorgan-1601 (stem explants).

Kinetin (mg L^−1^)	IAA (mg L^−1^)	Weight (g)	Callus (%)	Colour	Texture	Embryo stage	Shoot/plant
0	0	0.064^c^ ± 0.001	12.00^c^ ± 0.82	Green	CO	PE	—
0.5	0	0.337^b^ ± 0.019	11.25^c^ ± 0.68	Green	CO	GL	—
1	0	0.133^bc^ ± 0.014	35.00^bc^ ± 1.24	Light G.	FR	GL	—
1.5	0	0.312^b^ ± 0.021	73.75^ab^ ± 2.54	Cream	FR	PE	—
2	0	0.192^bc^ ± 0.018	23.75^bc^ ± 1.06	Light G.	CO	PE	—
0	0.5	0.088^c^ ± 0.002	41.25^bc^ ± 1.32	Light G.	FR	CT	10
0.5	0.5	0.156^bc^ ± 0.012	68.33^b^ ± 1.95	Green	CO	GL	—
1	0.5	1.083^a^ ± 0.082	51.67^b^ ± 1.75	Cream	FR	GL	—
1.5	0.5	0.155^bc^ ± 0.011	61.67^b^ ± 1.84	Light G.	FR	GL	—
2	0.5	0.450^b^ ± 0.028	68.33^b^ ± 1.94	Light G.	FR	CT	—
0	1	0.032^c^ ± 0.001	6.25^c^ ± 0.14	Green	CO	PE	—
0.5	1	0.842^ab^ ± 0.057	63.33^b^ ± 1.65	Light G.	FR	GL	—
1	1	0.502^b^ ± 0.023	76.25^ab^ ± 2.21	Light G.	CO	GL	—
1.5	1	0.089^c^ ± 0.002	71.25^ab^ ± 2.15	Green	CO	CT	3
2	1	0.122^bc^ ± 0.009	40.00^bc^ ± 1.41	Light G.	FR	PE	—
0	1.5	0.203^bc^ ± 0.014	77.50^ab^ ± 2.98	Light G.	FR	GL	—
0.5	1.5	1.003^a^ ± 0.086	100.0^a^ ± 3.21	Light G.	FR	CT	7
1	1.5	0.187^bc^ ± 0.019	57.50^b^ ± 1.65	Green	CO	CT	2
1.5	1.5	0.430^b^ ± 0.028	92.50^a^ ± 3.02	Green	CO	CT	8
2	1.5	0.819^a^ ± 0.068	78.33^ab^ ± 2.68	Green	CO	GL	—
0	2	0.077^c^ ± 0.005	30.22^bc^ ± 1.54	Green	CO	PE	—
0.5	2	0.771^ab^ ± 0.024	76.25^ab^ ± 2.45	Light G.	FR	GL	—
1	2	0.432^b^ ± 0.035	73.75^ab^ ± 2.42	Green	CO	CT	—
1.5	2	1.076^a^ ± 0.098	87.50^a^ ± 2.68	Light G.	FR	CT	—
2	2	0.176^bc^ ± 0.006	95.00^a^ ± 3.47	Green	CO	CT	—

The means of the populations with the same small letters were not significantly different as per Duncan's multirange test at P < 0.05.

CO: compact, FR: friable, PE: preembryo, GL: globular, CT: cotyledon, and G: green.

**Table 2:** Effect of Kinetin and IAA on mean weight and callus percentage of three *O. viciifolia* varieties: Golpaygan-181, Orumieh-1763, and Gorgan-1601 (leaf explants).

Kinetin (mg L^−1^)	IAA (mg L^−1^)	Weight (g)	Callus (%)	Colour	Texture	Embryo stage	Shoot/plant
0	0	0.046^c^ ± 0.002	26.67^bc^ ± 0.94	Green	CO	PE	—
0.5	0	0.337^b^ ± 0.028	55.00^b^ ± 1.45	Green	CO	GL	—
1	0	0.198^bc^ ± 0.019	86.67^a^ ± 2.35	Light G.	FR	GL	—
1.5	0	0.731^ab^ ± 0.034	97.50^a^ ± 3.58	Cream	FR	GL	—
2	0	0.292^b^ ± 0.016	52.50^b^ ± 1.98	Light G.	FR	PE	—
0	0.5	0.219^b^ ± 0.024	70.02^ab^ ± 2.14	Light G.	CO	CT	15
0.5	0.5	0.142^bc^ ± 0.09	68.33^ab^ ± 2.35	Light G.	FR	GL	—
1	0.5	0.639^ab^ ± 0.034	85.06^a^ ± 3.24	Cream	FR	GL	—
1.5	0.5	0.484^b^ ± 0.042	85.20^a^ ± 3.05	Light G.	CO	GL	—
2	0.5	0.121^bc^ ± 0.008	70.47^ab^ ± 2.45	Cream	FR	CT	—
0	1	0.179^bc^ ± 0.006	26.25^bc^ ± 0.98	Green	CO	PE	—
0.5	1	0.489^b^ ± 0.025	73.33^ab^ ± 2.45	Light G.	FR	GL	—
1	1	0.291^b^ ± 0.018	85.51^a^ ± 2.87	Light G.	CO	CT	—
1.5	1	0.163^bc^ ± 0.012	78.75^ab^ ± 2.34	Green	CO	CT	6
2	1	0.217^bc^ ± 0.015	68.33^ab^ ± 2.17	Light G.	FR	GL	—
0	1.5	0.174^bc^ ± 0.016	82.50^ab^ ± 3.78	Light G.	FR	CT	14
0.5	1.5	0.826^ab^ ± 0.057	100.0^a^ ± 3.45	Light G.	FR	CT	7
1	1.5	0.311^b^ ± 0.027	73.75^ab^ ± 3.15	Green	CO	CT	2
1.5	1.5	0.724^ab^ ± 0.065	96.25^a^ ± 3.54	Green	CO	GL	—
2	1.5	1.182^a^ ± 0.102	76.67^ab^ ± 2.97	Green	CO	GL	—
0	2	0.051^c^ ± 0.002	11.25^c^ ± 0.54	Green	CO	PE	18
0.5	2	1.015^a^ ± 0.098	100.0^a^	Light G.	FR	CT	—
1	2	0.288^b^ ± 0.017	92.50^a^ ± 2.54	Green	CO	GL	—
1.5	2	1.351^a^ ± 0.114	95.00^a^ ± 2.58	Light G.	FR	CT	—
2	2	0.266^b^ ± 0.016	88.33^a^ ± 2.41	Green	CO	GL	—

The means of the populations with the same small letters were not significantly different as per Duncan's multirange test at P < 0.05.

CO: compact, FR: friable, PE: preembryo, GL: globular, CT: cotyledon, G: green.

**Table 3:** Effect of storage durations and soil substrates on mean synthetic seed germination of control condition^∗^.

Varieties	Nonsterilized soil (%)	Sterilized soil (%)	Storage at 4°C		
			15 days	30 days	45 days
Leaf					
Golpaygan-181	10.25 ± 0.78	3.21 ± 0.12	6.24 ± 0.59	0.98 ± 0.08	—
Orumieh-1763	9.65 ± 0.67	4.87 ± 0.23	5.14 ± 0.46	0.35 ± 0.02	—
Gorgan-1601	6.23 ± 0.46	1.29 ± 0.11	3.24 ± 0.21	—	—
Stem					
Golpaygan-181	24.68 ± 1.35	8.97 ± 0.68	9.87 ± 0.12	3.54 ± 0.01	—
Orumieh-1763	23.54 ± 1.42	5.24 ± 0.58	6.74 ± 0.06	1.87 ± 0.01	—
Gorgan-1601	19.74 ± 1.28	3.66 ± 0.19	3.34 ± 0.02	—	—

*Control condition: sodium alginate + MS medium.

**Table 4:** The responses of multiple shoots derived from encapsulated seeds on MS medium supplemented with different auxins and cytokinins concentrations.

MS + hormones (mg L^−1^)		Observations (%)				Number	
	BAP	Shoot	Callus	Necrosis	Root	Root/plant	Shoot/plant
Control		NR	86.47	12.24	NR	—	—
IBA							
1	0.5	72.24 ± 3.24	22.48 ± 1.12	2.34 ± 0.15	NR	—	18
2	0.5	48.76 ± 1.57	33.37 ± 1.57	16.66 ± 1.10	3.23 ± 0.24	1	12
0.5	—	NR	23.24 ± 1.68	75.94 ± 2.68	NR	—	—
1	—	NR	78.23 ± 2.87	5.35 ± 0.24	12.84 ± 1.25	2	—
0.25	0.25	24.26 ± 1.14	12.35 ± 0.27	52.95 ± 1.68	NR	—	14
2	—	NR	42.35 ± 1.22	32.95 ± 1.27	18.24 ± 1.36	2	—
NAA							
1	0.5	NR	NR	12.48 ± 0.95	82.35 ± 2.68	3	—
0.5	—	21.35 ± 1.24	72.14 ± 3.07	5.87 ± 0.14	NR	—	7
0.25	—	NR	10.24 ± 0.68	48.41 ± 1.26	42.68 ± 1.63	2	—
0.5	—	NR	28.35 ± 1.34	61.84 ± 2.65	10.24 ± 1.15	—	—
2	—	NR	9.87 ± 0.36	35.24 ± 1.14	58.95 ± 1.74	—	—
IAA							
0.25	0.25	6.24 ± 0.24	46.23 ± 1.84	2.45 ± 0.14	47.68 ± 1.36	4	4
1	0.25	NR	66.24 ± 2.16	32.54 ± 1.36	NR	—	—
2	0.5	NR	75.62 ± 3.27	12.87 ± 1.08	NR	—	—
1	0.5	NR	72.32 ± 3.16	15.84 ± 1.24	NR	—	—
1	0.25	12.35 ± 0.47	32.24 ± 1.47	10.23 ± 0.84	46.98 ± 1.42	2	6
2	—	NR	58.62 ± 1.88	42.32 ± 2.15	NR	—	—
1	—	NR	42.35 ± 1.65	48.95 ± 2.06	NR	—	—
Half strength							
1 mg/L IBA	—	NR	42.65 ± 2.14	10.25 ± 1.02	48.01 ± 1.55	3	—
1 mg/L IAA	—	78.95 ± 2.57	NR	12.52 ± 1.24	10.65 ± 0.84	1	11
1 mg/L NAA	—	NR	23.65 ± 1.06	24.51 ± 1.28	52.62 ± 2.47	2	—

NR = no response was obtained.

**Table 5:** Acclimatization of plantlets of *O. viciifolia* in different soil substrates.

Methods	Observations	Survival rate (%)
Autoclaved black soil + 1/2 MS	Most plantlets were weak with low vigour	45%
Non-autoclaved black soil	Most plantlets became weak after 3 weeks	65%
Non-autoclaved red soil	Normal growth but some plots contaminated due to high humidity	72%
Non-autoclaved black soil : red soil at ratio 1 : 1	The best situation of water adjustment, normal growth	98%

## References

[1] Chen WH, Davey MR, Power JB, Cocking EC (1988). Control and maintenance of plant regeneration in sugarcane callus cultures. *Journal of Experimental Botany*.

[2] Merkle SA, Parrot WA, Flinn BS, Thorpe TA (1995). Morphogenetic aspects of somatic embryogenesis. *In-Vitro Somatic Embryogenesis in Plants*.

[3] Siong PK, Mohajer S, Taha RM (2012). Production of artificial seeds derived from encapsulated *in vitro* micro shoots of cauliflower, Brassica oleracea var. botrytis. *Romanian Biotechnological Letters*.

[4] Wang GR, Qi NM (2010). Influence of mist intervals and aeration rate on growth and second metabolite production of *Pseudostellaria heterophylla* adventitious roots in a siphon-mist bioreactor. *Biotechnology and Bioprocess Engineering*.

[5] Bapat VA, Rao PS (1990). *In vivo* growth of encapsulated axillary buds of mulberry (*Morus indica* L.). *Plant Cell, Tissue and Organ Culture*.

[6] Sancak C (1999). *In vitro* micropropagation of sainfoin (*Onobrychis viciifolia* Scop.). *Turkish Journal of Botany*.

[7] Karamian R, Ranjbar M (2008). Plant regeneration from *Onobrychis subnitens* Bornm. hypocotyl explants via somatic embryogenesis and organogenesis. *Acta Biologica Cracoviensia Series Botanica*.

[8] Cha-um S, Mosaleeyanon K, Supaibulwatana K, Kirdmanee C (2003). A more efficient transplanting system for Thai neem (*Azadirachta siamensis* Val.) by reducing relative humidity. *Asian Science*.

[9] Talbott LD, Rahveh E, Zeiger E (2003). Relative humidity is a key factor in the acclimation of the stomatal response to CO_2_. *Journal of Experimental Botany*.

[10] Premkumar A, Mercado JA, Quesada MA (2001). Effects of *in vitro* tissue culture conditions and acclimatization on the contents of Rubisco, leaf soluble proteins, photosynthetic pigments, and C/N ratio. *Journal of Plant Physiology*.

[11] Özcan S, Sevimay CS, Yildiz M, Sancak C, Özgen M (1996). Prolific shoot regeneration from immature embryo explants of sainfoin (*Onobrychis viciifolia* Scop.). *Plant Cell Reports*.

[12] Mohajer S, Taha RM, Khorasani A, Yaacob JS (2012). Induction of different types of callus and somatic embryogenesis in various explants of Sainfoin (*Onobrychis viciifolia*). *Australian Journal of Crop Science*.

[13] Müller C, Riederer M (2005). Plant surface properties in chemical ecology. *Journal of Chemical Ecology*.

[14] Riederer M, Schreiber L (2001). Protecting against water loss: analysis of the barrier properties of plant cuticles. *Journal of Experimental Botany*.

[15] Burghardt M, Riederer M, Riederer M, Müller C (2006). Cuticular transpiration. *Biology of the Plant Cuticle*.

[16] Kerstiens G (1996). Cuticular water permeability and its physiological significance. *Journal of Experimental Botany*.

[17] Fürstner R, Barthlott W, Neinhuis C, Walzel P (2005). Wetting and self-cleaning properties of artificial superhydrophobic surfaces. *Langmuir*.

[18] Gorb E, Haas K, Henrich A, Enders S, Barbakadze N, Gorb S (2005). Composite structure of the crystalline epicuticular wax layer of the slippery zone in the pitchers of the carnivorous plant *Nepenthes alata* and its effect on insect attachment. *Journal of Experimental Biology*.

[19] Holmes MG, Keiller DR (2002). Effects of pubescence and waxes on the reflectance of leaves in the ultraviolet and photosynthetic wavebands: a comparison of a range of species. *Plant, Cell and Environment*.

[20] Pfündel EE, Agati G, Cerovic GZ, Riederer M, Müller C (2006). Optical properties of plant surfaces. *Biology of the Plant Cuticle*.

[21] Ren L-Q, Wang S-J, Tian X-M, Han Z-W, Yan L-N, Qiu Z-M (2007). Non-smooth morphologies of pica1 plant leaf surfaces and their anti-adhesion effects. *Journal of Bionic Engineering*.

[22] Brewer CA, Smith WK, Vogelmann TC (1991). Functional interaction between leaf trichomes, leaf wettability and the optical properties of water droplets. *Plant Cell Environment*.

[23] Gupta PK, Durzan DJ (1987). Biotechnology of somatic polyembryogenesis and plantlet regeneration in loblolly pine. *Bio/Technology*.

[24] George EF, Sherrington PD (1984). *Plant Propagation by Tissue Culture*.

[25] Maruyama E, Ishii K, Kinoshita I (1998). Alginate encapsulation technique and cryogenic procedures for long-term storage of the tropical forest tree *Guazuma crinita* mart. *in vitro* cultures. *Japan Agricultural Research Quarterly*.

[26] Ipekci Z, Gozukirmizi N (2003). Direct somatic embryogenesis and synthetic seed production from *Paulownia elongata*. *Plant Cell Reports*.

[27] Alam MZ, Haider SA, Islam R, Joader OL (1995). High frequency *in vitro* regeneration in sugarcane. *Sugarcane*.

[28] Islam R, Haider SA, Alam MA, Joarder OI (1996). High frequency somatic embryogenesis and plant regeneration in sugarcane. *Rice Biotechnoligy Quareterly*.

[29] Bargel H, Neinhuis C (2005). Tomato (*Lycopersicon esculentum* Mill.) fruit growth and ripening as related to the biomechanical properties of fruit skin and isolated cuticle. *Journal of Experimental Botany*.

[30] Gates DM, Lange OL, Kappen L, Schulze ED (1976). Energy exchange and transpiration. *Ecolog Stud. Water and Plant Life*.

[31] Mott KA, Gibson AC, O'Lerary JW (1982). The adaptive significance of amphistomatic leaves. *Plant Cell Environment*.

[32] Jeffree CE, Riederer M, Müller C (2006). The fine structure of the plant cuticle. *Biology of the Plant Cuticle*.

[33] Kolattukudy PE, Scheper T (2001). Polyesters in higher plants. *Advances in Biochemical Engineering/ Biotechnology*.

[34] Gibson AC (1996). *Structure-Function Relations of Warm Desert Plants*.

[35] Martin C, Glover BJ (2007). Functional aspects of cell patterning in aerial epidermis. *Current Opinion in Plant Biology*.

[36] Koch K, Bhushan B, Barthlott W (2009). Multifunctional surface structures of plants: an inspiration for biomimetics. *Progress in Materials Science*.

[37] Eller BM (1985). Epidermis und spektrale Eigenschaften pflanzlicher oberflachen. *Berichte der Deutschen Botanischen Gesellschaft*.

[38] Poppinga S, Koch K, Bohn HF, Barthlott W (2010). Comparative and functional morphology of hierarchically structured anti-adhesive surfaces in carnivorous plants and kettle trap flowers. *Functional Plant Biology*.

[39] Koch K, Bhushan B, Barthlott W (2009). Multifunctional surface structures of plants: an inspiration for biomimetics. *Progress in Materials Science*.

[40] Fauteux F, Rémus-Borel W, Menzies JG, Bélanger RR (2005). Silicon and plant disease resistance against pathogenic fungi. *FEMS Microbiology Letters*.

